# Fulminant Hepatitis A and E Co-infection Leading to Acute Liver Failure: A Case Report

**DOI:** 10.7759/cureus.38101

**Published:** 2023-04-25

**Authors:** Haider Malik, Hamza Malik, Muskan Uderani, Mefthe Berhanu, Cuauhtemoc Jeffrey Soto, Faraz Saleem

**Affiliations:** 1 Medical School, Shifa Tameer-E-Millat University Shifa College of Medicine, Islamabad, PAK; 2 Medical School, Foundation University Medical College, Rawalpindi, PAK; 3 Internal Medicine, Liaquat University of Medical and Health Sciences, Hyderabad, PAK; 4 Health Science, University of Texas Health Science Center at Houston, Houston, USA; 5 Research and Development, Universidad Juarez del Estado de Durango, Durango, MEX; 6 Internal Medicine, California Institute of Behavioral Neurosciences & Psychology, Fairfield, USA; 7 Internal Medicine, Akhtar Saeed Medical and Dental College, Lahore, PAK

**Keywords:** prompt management, early diagnosis, liver transplant, fhf, co-infection, hepatitis e, hepatitis a, fulminant hepatitis, acute liver failure

## Abstract

Acute liver failure (ALF) is a severe clinical condition with a high mortality rate. Although several factors can cause ALF, viral hepatitis remains one of the leading causes. Hepatitis A virus (HAV) and hepatitis E virus (HEV), which typically cause self-limiting acute disease, are rare but emerging causes of ALF, especially when both viruses infect the same individual. Both of these hepatotropic viruses share an enteric route and are most commonly transmitted through the fecal-oral route. The impact of HAV/HEV co-infection on acute hepatitis prognosis is not entirely understood, but dual infection can further exacerbate liver damage, leading to fulminant hepatic failure (FHF) with a higher mortality rate than a single virus infection.

Here, we present a case of a 32-year-old male with no prior liver disease who presented to the emergency department with a two-week history of jaundice, abdominal pain, and hepatomegaly. Upon admission, he was disoriented with grade 2 encephalopathy. After a thorough investigation, co-infection with hepatitis A and E was identified as the primary cause of his ALF. The patient underwent intensive medical treatment and interventions, including dialysis. Unfortunately, the patient's survival was not possible due to the absence of availability of a transplanted organ, which is currently the only definitive treatment option.

This case report underscores the significance of prompt diagnosis, timely intervention, and the accessibility of transplantation in the survival of liver failure, as it remains the sole definitive treatment for acute liver failure. Moreover, it provides a concise overview of the current literature on fulminant co-infection of HAV and HEV, including epidemiology, clinical characteristics, pathogenesis, diagnosis, treatment, and risk factors associated with co-infection of hepatitis A and E and their role in causing ALF. It also highlights the significance of identifying high-risk populations and implementing appropriate prevention and control measures such as vaccination, practising good hygiene and sanitation, and avoiding the consumption of contaminated food and water.

## Introduction

Acute liver failure (ALF) is a life-threatening complication of acute viral hepatitis, marked by the rapid deterioration of liver function. Acute liver failure can have high mortality rates if not properly treated immediately with intensive care, targeted treatment plans based on etiology or liver transplantation. Viral hepatitis, particularly hepatitis A virus (HAV) and hepatitis E virus (HEV), as well as drugs such as paracetamol, are among the primary causes of acute viral hepatitis globally. Even though HAV and HEV infections tend to resolve on their own, they can sometimes progress into fulminant hepatic failure (FHF), especially for individuals with preexisting liver conditions or pregnant women. Co-infection with both HAV and HEV infections is a rare but emerging clinical entity which may lead to severe liver injury and ALF [1-2].

Hepatitis A and E account for an enormous percentage of cases worldwide of acute liver failure. Bernal and Wendon have reported that HAV) and HEV) are major causes of ALF worldwide, with a mortality rate exceeding 50%, especially in developing countries. In one study conducted in India, the incidence of ALF related to HAV and HEV was found to be as high as 44% [3].

The World Health Organization (WHO) in 2020, estimated that hepatitis A causes approximately 1.4 million infections and approximately 7,000 deaths each year, while hepatitis E causes 20 million infections, 3.3 million symptomatic cases, and 44,000 fatalities annually [1-2]. Its case-fatality rate ranges between 0.5-2% with cases co-infected with hepatitis A or E being highly contagious regardless of proper sanitation and hygiene facilities available suggesting efficient interpersonal transmission [2-3].

Acute liver failure requires a multifaceted approach to management, which involves addressing the underlying cause, monitoring for progression, treating complications, managing hemodynamic stability, and providing nutritional support. Only 40% of patients with acute liver failure achieve spontaneous recovery, necessitating liver transplantation in many cases. Predicting the need for transplantation depends on various prognostic factors, including the degree of encephalopathy, underlying cause, age, and prognostic models like the Model for End-stage Liver Disease (MELD). The overall survival rates for acute liver failure patients treated without liver transplantation are over 60%. Still, the prognosis of those awaiting transplantation varies and is unpredictable. In the US, 67% of acute liver failure patients survived, and after transplantation, one-year survival was around 80%, with deaths mostly due to neurologic complications or sepsis within three months of transplantation [1].

Therefore, it is crucial to have early diagnosis, appropriate management, and timely referral to a transplant centre for patients with ALF caused by hepatitis A or E. This case report aims to summarize the current literature on fulminant co-infection of HAV and HEV, including its epidemiology, clinical features, pathogenesis, diagnosis, treatment, and risk factors associated with co-infection of hepatitis A and E and their role in causing ALF. It also highlights the importance of identifying high-risk populations and designing appropriate prevention and control strategies to reduce the burden of hepatitis A and E-related ALF.

## Case presentation

A 38-year-old Asian man with a two-week history of abdominal discomfort, lethargy, and jaundice arrived at the emergency room of the tertiary care hospital. Two days ago, he began to experience altered mental status and was disoriented in time and space. According to his family, the patient had no notable past medical history and denied having recently travelled or been among sick people. Additionally, the patient's wife stated that her husband is not an alcoholic and denied any recent intake of drugs by him. The patient was discovered to have severe jaundice, with yellow discoloration of his sclera and skin, upon physical examination. His abdomen was tender, and his liver was palpable. He displayed grade 2 hepatic encephalopathy and was noticeably disoriented and confused. His Glasgow Coma Scale (GCS) score was 10.

The results of the laboratory tests showed that the patient's liver function levels were raised. These included an aspartate transaminase (AST) of 2,100 U/L (8-48 U/L), alanine transaminase (ALT) of 2,200 U/L (7-55 U/L), total bilirubin of 28 mg/dL (0.3-1.2 mg/dL), and an International Normalized Ratio (INR) of 2.4. With immunoglobulin M (IgM) antibody titres for the hepatitis A (HAV) and hepatitis E (HEV) virus of 1:2560 and 1:640, respectively, the serologic test for HAV and HEV viruses yielded a positive result. This proved the co-infection of both viruses. Polymerase chain reactions (PCR) detected HEV ribonucleic acid (RNA) in the patient's serum, indicating viral reproduction that was active (Table 1).

**Table 1:** Lab parameters of the patient at the time of admission.

Lab Parameter	Result	Normal Range
Haemoglobin (Hb)	14 g/dL	13.5 - 17.5 g/dL
White blood cell count (WBC)	18000/μL	4000 - 11000/μL
Alanine aminotransferase (ALT)	2200 U/L	10 - 40 U/L
Aspartate aminotransferase (AST)	2100 U/L	10 - 35 U/L
Bilirubin	28 mg/dL	0.2 - 1.2 mg/dL
Ammonia	59 μmol/L	11 - 35 μmol/L
International Normalized Ratio (INR)	2.4	0.9 - 1.1
Creatinine (Cr)	1.1 mg/dL	0.6 - 1.2 mg/dL
Glucose (fasting)	75 mg/dL	70 - 110 mg/dL
Sodium (Na)	128 mEq/L	135 - 145 mEq/L
Potassium (K)	3.2 mEq/L	3.5 - 5.0 mEq/L
Hepatitis A virus IgM Antibody Titer	1:2560	N/A
Hepatitis E virus IgM Antibody Titer	1:640	N/A

The patient was admitted to the hospital with a diagnosis of acute liver failure (ALF) due to hepatitis A and E co-infection. Upon admission, the patient was shifted to the intensive care unit (ICU) and started on supportive therapy. This included intravenous fluids to maintain hydration, electrolyte replacement, and total parenteral nutrition (TPN) to provide essential nutrients to his body. Serial laboratory tests were done, including six-hourly monitoring of his coagulation parameters including prothrombin time (PT) and platelets, complete blood counts (CBC), metabolic panels, and arterial blood gases (ABG). His liver function tests, including bilirubin, ALT, and AST, were also done every 12 hours to assess the severity of his liver disease. His Model for End-stage Liver Disease (MELD) score was 28, based on his lab parameters, predicting a spontaneous recovery. He was also referred to a transplant centre for a possible liver transplant in case of worsening of his symptoms.

He was started on antiviral therapy with ribavirin, given the evidence of active HEV replication. He was also given lactulose, about 20-30 g every 2 hours initially, but it showed no improvement, so it was tapered off. His hypoglycemia and electrolytes were corrected accordingly. There was also evidence of increased intracranial pressure (ICP), confirmed through cranial Doppler ultrasound, so a bolus of mannitol (0.5 g/Kg) was given to reduce the intracranial pressure. Unfortunately, despite all the efforts made there was not a single sign of improvement, and the patient's condition further started worsening. Regardless of his initial MELD score of 28 at the admission, which initially suggested a good prognosis, the patient's condition deteriorated rapidly. His ammonia and bilirubin levels started rising and he began to show signs of severe grade 4 encephalopathy. The patient's deteriorating encephalopathy and hyperammonemia prompted the initiation of dialysis; however, the medical team's intensive measures failed to improve the patient's condition, ultimately resulting in his demise. Unfortunately, the unavailability of a transplant donor despite taking early measures for the transplant rendered his survival exceedingly improbable and resulted in the patient's demise within 48 hours after admission.

## Discussion

Acute liver failure (ALF) is the sudden and severe impairment of liver function that occurs in patients without cirrhosis or previous liver disease. This is manifested as encephalopathy and a reduced capacity for synthesis. An international normalized ratio (INR) >=1.5 indicates acute liver failure. There is some variation in the time period that separates acute liver disease from chronic liver disease across studies. However, an illness lasting less than 26 weeks is commonly accepted as a threshold [1]. In the absence of immediate intensive care, specific treatments, or liver transplantation, it is associated with a high mortality rate [2]. ALF is caused by a variety of factors, including drug overdoses (including acetaminophen), viral infections, ischemia, and others. Although a complete recovery of liver function is possible in cases where ALF does not result in death, accurately predicting the likelihood of mortality can be difficult [2].

Acute liver disease is associated with several viruses, including hepatitis A (HAV). It's estimated that 0.35% of patients with hepatitis A develop acute liver disease. Hepatitis E (HEV) infection is a major cause of liver disease in countries like Russia, Pakistan, and India. However, overall, the hepatitis E case fatality rate is 0.5-3%. The case fatality rate increases significantly when hepatitis A or E is co-infected [3-4].

According to the World Health Organization (WHO), in 2020, it was estimated that HAV infections caused 3.4 million symptomatic cases and 44,000 deaths [1-2,5]. HAV is transmitted primarily by fecal-oral contact between people, although contaminated water or food can also cause transmission. HEV, on the other hand, is transmitted primarily by contaminated food or water. HAV is more likely to be contracted by those who engage in oral-anal sexual activity, regardless of their gender or orientation [5]. This includes homosexual men. Individuals who have used contaminated intravenous drugs or blood products are at a higher risk of infection. The virus can be found in the stool up to two weeks prior to the onset of symptoms and even for a few days afterwards. Some individuals continue excreting the virus for many weeks. The rate of transmission among close contacts of a case remains high despite adequate sanitation and hygiene, which indicates efficient interpersonal transmission. The pattern and rate of transmission are primarily influenced by socioeconomic variables. Lower infection rates are observed in areas with smaller families, better sanitation facilities, and greater access to clean water [5-8].

HEV1 and HEV2 are similar to HAV, in that they are endemic and transmitted by fecal or oral routes in low-income countries. HEV3 and 4 are zoonotic viruses that can infect many mammalian species. Pigs, however, are their primary hosts. Humans are primarily infected by the virus through eating meat that has been infected. According to the World Health Organization, HEV causes approximately 20 million infections per year. This leads to approximately 3.3 million symptomatic cases as well as 44,000 deaths. HAV and HEV epidemiology is affected by the genotype, which is determined geographically. Children in high-income areas with low endemicity like Australia, Japan and Western Europe have lower HAV exposure rates. The adult population of these regions has a lower proportion of anti-HAV antibodies. This population has a greater risk of contracting HAV. Significant outbreaks can occur, particularly among high-risk populations such as homeless individuals, recreational drug users and men who have had sex with other men [5-8].

Dual infection, also known as co-infection, is a phenomenon that has been documented in many developing and developed countries. HAV/HEV co-infections have been documented in reports from developing countries, such as India, Mexico, Kenya, Bangladesh, and Egypt. In India, HAV/HEV coinfections were found to occur at a rate of 1.3%. The HEV infection rate was 16.1% and the HAV infection rate was 12.6%. In comparison to HEV and HAV mono-infections, dual infection cases had more abnormal liver functions. HEV/HBV was the most prevalent haptotropic double infection in India among viral hepatitis, while HAV/HEV was more common. In India, the mode of transmission for HAV and HEV is fecal-oral. In Egypt, 26% of 268 cases of acute viral hepatitis in children were confirmed to have HAV/HEV double infection. In Venezuela, the proportion of acute hepatitis cases with HAV/HEV double infection (31%) was higher than that of HEV mono-infection (29%). An HAV/HEV double infection has been reported in Mexico. The HEV strains circulating are of genotype 1. Five patients (10.87%) of 46 with acute hepatitis in China were HAV/HEV double-infected. HEV/HAV dual infection was documented in Italy in men who have sex with men (MSM). In Israel, high anti-HAV antibodies were observed in HEV seropositive haemophiliac patients, suggesting HAV/HEV dual infection [6].

Numerous studies have shown that dual infection with HEV and HAV may result in serious outcomes. Arora et al. conducted an investigation to determine the cause of ALF among children. They found that dual infection with HAV and HEV accounted for the majority of FHF cases in children (20.45%), of which three were fatal [9]. Dual infection with HAV and HEV caused a higher incidence of ALF than either virus alone. The rates were 9% for HAV and 13.6% for HEV. An HAV/HEV double infection can also lead to complications like acalculous cystitis and hepatic neuropathy, which are the worst possible outcomes. As evidenced by liver abnormalities and liver function tests, other studies support the conclusion that HEV/HAV double infection can cause a severe disease course [6,9-10]. Similarly, Paul et al. reported a higher mortality rate for HEV infections in pregnant women. This was especially true of those who were also co-infected by hepatotropic viruses such as HBV and HAV [10].

Hepatitis A, an infection caused by the hepatitis A virus, can present in various clinical presentations. These range from asymptomatic hepatitis to fulminant liver disease. The course of the disease is usually more severe in older people, whereas very young children tend to be asymptomatic. The infection is divided into a prodromal and icteric phase, the latter of which includes significant jaundice and pruritus as well as fever, diarrhea, weight loss, and malaise. Acute liver failure, while rare, can happen in patients with chronic HBV and hepatitis C virus (HCV) infections. Liver function tests (LFT) reveal a cholestasis-like pattern marked by bilirubin, alkaline phosphatase (ALP), and a mild or moderate increase in ALT [4,10-11]. Atypical cholestatic disease can occur in some patients. A relapse of hepatitis A is possible within six months after the initial infection for 10% of patients. Patients with cholestatic liver disease are usually treated adequately with supportive care, and the majority of patients fully recover after a relapse [5,12]. Approximately 0.3% of individuals with hepatitis A (HAV), though the rate is heavily influenced by their age, experience acute liver failure. The case fatality rates range from 0.1% to 0.3% among children and adults younger than 40 years. The case fatality rate is 1.8% in adults older than 49 [5,13]. It is not clear what factors contribute to the rare progression of HAV to ALF. Host factors such as age and mild liver damage could play a role [2,14]. In addition, viral factors such as low viral loads and a high rate of substitutions within the 5' untranslated area of the viral genome may increase the likelihood of a severe case [2,15].

The host's immune response to HAV causes hepatic injury. Viral replication takes place in the hepatocyte's cytoplasm. Hepatocellular injury and destruction of infected cells are mediated by CD8+ T-lymphocytes that are HAV-specific and restricted to human leukocyte antigen (HLA). Interferon-gamma seems to play a key role in the clearance of infected liver cells. Severe hepatitis is linked to an excessive host response (denoted by a marked decrease in the circulation of HAV ribonucleic acids (RNA)) during acute infection [7].

Hepatitis E Virus infection, a self-limiting, acute illness, typically lasts 4-6 weeks and is characterized by symptoms like jaundice and fatigue. It can also cause nausea and vomiting, as well as abdominal pain. In developing countries where HEV1 or HEV2 is predominant, young adults are the most affected. However, in more resource-rich settings, older men are the ones who are primarily affected by HEV-3 or HEV-4. Acute hepatitis E is associated with pre-existing liver diseases and other risk factors such as diabetes and alcohol abuse, but liver failure progression is rare. Individuals with pre-existing chronic liver disease may be at increased risk for acute liver failure. HEV, despite its clinical heterogeneity in certain European countries, is the most common cause of viral liver disease [5,16]. Children under three and vulnerable groups are at a higher risk of death, particularly during pregnancy. In this third trimester, 33% of pregnant women can develop fulminant liver failure [5,17]. HEV-related deaths are primarily caused by liver failure and obstetric complications such as haemorrhage or eclampsia [17]. Vertical transmission, which is very common, poses a significant risk to the fetus as well as the neonate. There are also increased risks of intrauterine deaths, stillbirths, preterm births, and low birth weight (LBW). The fetal mortality rate is about 33%. This includes those who die due to maternal death. Neonatal mortality is only around 8% [18]. Hepatitis E, which is a virus, accounts for 20-40% of all cases of acute liver failure in developing countries. In Western countries, liver damage caused by HEV is only now being recognized as an imported infection. According to this view, population-based studies in America show that hepatitis E causes ALF on a rare basis [2,19].

Patients with prodromal symptoms, such as nausea, vomiting, abdominal pain, jaundice, or elevated serum levels of aminotransferase, should be suspect of an acute HAV infection, especially if they have known risk factors of hepatitis A. Anti-HAV serum Immunoglobulin M (IgM) antibodies can be detected to confirm the diagnosis. Serum IgM anti-HAV antibodies are detected at the time symptoms begin to peak in the acute phase or early convalescent stage and persist for three to six months. Serum IgM antibodies are present for the entire duration of relapsing liver disease in patients [5,7].

The lack of a standard assay complicates the diagnosis of acute HEV. There are many commercial kits for enzyme immunoassays (EIAs) that have high levels of variability. False positives and false negatives can be common. Anti-HEV IgM is the primary test, and it indicates recent HEV infections if IgM anti-HEV antibodies exist. Since no EIA test has a high degree of specificity, confirmatory tests should be done if the initial result is positive. Confirmatory tests can include an alternative anti-HEV IgM test, evidence of increasing anti-HEV IgG titres, or detection of HEV RNA in serum or stools. Repeat testing is recommended if the EIA test was negative but there are still suspicions of HEV infection. This should preferably be done using an HEV-RNA assay. Due to the high rate of false-negative antibody tests, immunocompromised patients with suspected HEV infection should be tested for their RNA [5-6,8].

IgM anti-HEV is detected early in the illness. It disappears in four to five months, but it may not be detected when the infection is acute. IgG anti-HEV is detected shortly after IgM and increases in titer from the acute phase to the convalescent stage. Some reports have detected IgG antibodies up to 14 years after the acute phase, but it is not clear how long they last. A booster effect from reinfection can't be ruled out. There may be differences in the assays for IgG anti-HEV antibodies, and IgM anti-HEV's sensitivity and specificity can also vary. HEV RNA is detectable in stool for up to 2 weeks before symptoms appear. Serum can be detected between two and six weeks following infection. It may persist for up to two weeks for those who have recovered from an acute infection. It can last for many years in people who have chronic infections. In most cases, the viremia lasts only a short time but can persist for years in chronic infections [5-6,8].

Most cases of hepatitis A or acute hepatitis E are self-limiting and recover spontaneously without the need for any specific treatment. However, patients who experience fulminant liver failure may require aggressive supportive therapy, and they could be candidates for liver transplantation [1,20]. The general management of patients with acute liver failure includes monitoring the liver for worsening, treating complications, and providing nutritional support. When possible, patients with acute liver failure need to be treated in an intensive care unit at a liver transplant center. The transfer to a liver transplant center should not delay the initiation of diagnostic tests or therapy. Serial laboratory tests can be used to track the progression of liver failure in a patient and monitor complications. Daily monitoring of serum aminotransferases, bilirubin, and metabolic panels is recommended. Monitoring should be done more frequently (three to four times daily) for coagulation parameters, a complete blood count (CBC), a metabolic panel, and arterial blood gases (ABG). Every six hours, we perform fingersticks to measure blood glucose. However, it is worth noting that only 40% of patients with this condition achieve spontaneous recovery, necessitating liver transplantation for many individuals [1,20-22].

The decision to proceed with transplantation hinges on the likelihood of the liver recovering independently, which can be predicted through several prognostic factors, such as the degree of encephalopathy, the underlying cause of the liver failure, patient age, and the use of prognostic models such as MELD. It is worth mentioning that the overall survival rates for patients receiving treatment for acute liver failure are high, with more than 60% of patients surviving without requiring a liver transplant [21-22]. However, the prognosis of those awaiting transplantation can vary significantly and is not always predictable. A study conducted in the United States involving 308 patients with acute liver failure revealed that 67% of them survived. After undergoing liver transplantation, the one-year survival rate was roughly 80% [1,21-22]. Notably, the majority of deaths occurring in patients who undergo transplantation are due to neurologic complications or sepsis and typically take place within three months of the transplant [1,20].

In order to prevent the spread of HAV, it is important to follow good hygiene and sanitation practices, conduct case investigations, trace contacts during outbreaks, and administer active and passive immune prophylaxis. To prevent infection, it is important to practice thorough handwashing and food handling. Close contacts who have been exposed to an infected individual may choose post-exposure prevention (PEP), such as intramuscular immune serum or vaccination, according to local guidelines. The U.S. Centers for Disease Control and Prevention (CDC) recommend that vaccinations be given to healthy adults between the ages of 12 months and 40 years, and immunoglobulin for individuals over 40. Immunoglobulin is also recommended for individuals who are immunocompromised, have chronic liver disease, or have other contraindications to vaccines. The CDC recommends that those who do not test positive for HAV be vaccinated. This is especially true for individuals who are at high risk of contracting HAV. These include intravenous drug users, men who have had a sexual relationship with men, as well as HIV, HBV, or HCV patients [2,4,7-8,20].

In regions where HEV1 and HEV2 are prevalent, preventive measures should encompass enhanced sanitation practices and the provision of safe potable water. In regions abundant in resources where zoonotic infections serve as the primary mode of transmission, the preparation of food, particularly pork, is a crucial preventive measure. Individuals with liver disease, immunosuppression, or other severe medical conditions are advised against consuming raw meat. Individuals who directly encounter boars or pigs and their byproducts are advised to utilize protective equipment and restrict their exposure [2,4,7-8,20].

Close contacts can also consider post-exposure prevention, including vaccination or intramuscular immunoglobulin, in accordance with local recommendations. In China, an effective HEV vaccine, HEV-239, has been available for several years. It offers long-term immunity against all HEV genotypes, but it is not yet licensed outside China [2,5,7-8,20].

## Conclusions

Fulminant co-infection of HAV) and HEV is a rare but emerging clinical entity that can lead to severe liver injury and ALF, particularly in regions where both HAV and HEV are endemic. Prompt diagnosis, timely intervention, and the accessibility of transplantation are crucial elements in the survival of liver failure, as it remains the sole definitive remedy for acute liver failure. Hence, it is imperative to mitigate the occurrence of fulminant co-infection by means of vaccination and public health interventions against HAV and HEV infections.
